# Sequential phototrophic–mixotrophic cultivation of oleaginous microalga *Graesiella* sp. WBG-1 in a 1000 m^2^ open raceway pond

**DOI:** 10.1186/s13068-019-1367-1

**Published:** 2019-02-11

**Authors:** Xiaobin Wen, Huanping Tao, Xinan Peng, Zhongjie Wang, Yi Ding, Yan Xu, Lin Liang, Kui Du, Aoqi Zhang, Caixia Liu, Yahong Geng, Yeguang Li

**Affiliations:** 1 CAS Key Laboratory of Plant Germplasm Enhancement and Specialty Agriculture, Wuhan Botanical Garden, Chinese Academy of Sciences, Wuhan, 430074 China; 2 University of Chinese Academy of Sciences, Beijing, 100049 China; 3 Present Address: Institute of Bioengineering, Zhengzhou Normal University, Zhengzhou, 450044 China; 4 Present Address: Sichuan Provincial Academy of Natural Resource Sciences, Chengdu, 610015 China

**Keywords:** Microalgae cultivation, *Graesiella*, Mixotrophic, Open raceway pond, SPMC

## Abstract

**Background:**

Microalgae are an important feedstock in industries. Currently, efforts are being made in the non-phototrophic cultivation of microalgae for biomass production. Studies have shown that mixotrophy is a more efficient process for producing algal biomass in comparison to phototrophic and heterotrophic cultures. However, cultivation of microalgae in pilot-scale open ponds in the presence of organic carbon substrates has not yet been developed. The problems are heterotrophic bacterial contamination and inefficient conversion of organic carbon.

**Results:**

Laboratory investigation was combined with outdoor cultivation to find a culture condition that favors the growth of alga, but inhibits bacteria. A window period for mixotrophic cultivation of the alga *Graesiella* sp. WBG-1 was identified. Using this period, a new sequential phototrophic–mixotrophic cultivation (SPMC) method that enhances algal biomass productivity and limits bacteria contamination at the same time was established for microalgae cultivation in open raceway ponds. *Graesiella* sp. WBG-1 maximally produced 12.5 g biomass and 4.1 g lipids m^−2^ day^−1^ in SPMC in a 1000 m^2^ raceway pond, which was an over 50% increase compared to phototrophic cultivation. The bacterial number in SPMC (2.97 × 10^5^ CFU ml^−1^) is comparable to that of the phototrophic cultivations.

**Conclusions:**

SPMC is an effective and feasible method to cultivate lipid-rich microalgae in open raceway ponds. Successful scale-up of SPMC in a commercial raceway pond (1000 m^2^ culture area) was demonstrated for the first time. This method is attractive for global producers of not only lipid-rich microalgae biomass, but also astaxanthin and β-carotene.

**Electronic supplementary material:**

The online version of this article (10.1186/s13068-019-1367-1) contains supplementary material, which is available to authorized users.

## Background

Microalgae are sources of many high value chemicals. *Arthrospira*, *Chlorella*, *Haematococcus*, and *Chaetoceros* have long been used in the health-care, cosmetic, and aquatic feed industries [1]. In recent years, microalgae have also been investigated as a potential material for waste carbon dioxide (CO_2_) fixation [2, 3] and biodiesel production [4, 5] due to their high solar energy utilization efficiency and unique cell compositions.

Algae biomass production is conventionally conducted in large open ponds [6]. Microalgae in the ponds grow phototrophically by harvesting sunlight and assimilating CO_2_ and water. The phototrophic growth of microalgae is technically feasible with open ponds and photo-bioreactors for commercial use; however, it has not yet been established to give satisfactory cell productivity in practice [6, 7]. To improve biomass and lipid productivity, phototrophic culture conditions have been modified in favor of cell growth and lipid accumulation. These conditions include illumination intensity [8], type and concentration of certain nutrition [9–11], type of reactor and mixing [4, 12]. Despite this, biomass yields are still lower than expected.

Currently, efforts are being made for the non-phototrophic cultivation of microalgae [13–16]. Studies have shown that many phototrophic microalgae can grow under heterotrophic or mixotrophic conditions [17]. Mixotrophic microalgae assimilate CO_2_ and organic carbon simultaneously, where light irradiation is also required [16]. Under such growth conditions, the energy needs for carbon reduction are derived both from organic carbon and harvested light [17]. Moreover, several studies on the microalgal energy metabolism showed that mixotrophic cultures resulted in higher energetic efficiency because the amount of energy dissipated was minimal [18, 19]. Therefore, mixotrophy is a more efficient process for producing algal biomass in comparison to phototrophic and heterotrophic cultures. This conclusion has been confirmed by many laboratory investigations [20–23]. Although mixotrophy has a great potential for efficient microalgae biomass production, the cultivation of microalgae in pilot-scale open ponds in the presence of organic carbon substrates has not yet been developed. Some technical obstacles, such as the inefficient conversion of organic carbon and heterotrophic bacterial contamination, need to be resolved before this technique can be applied to open raceway ponds.

Recently, it was suggested that the distinct dynamic properties of algal and bacterial growth and its nutrient demands should be exploited to favor the growth of alga over that of bacteria [16]. In this study, we focus on the window period immediately after the nitrate depletion of a culture, during which the algal cells only grow on intracellular nitrogen. If acetate is supplemented during this window period, mixotrophic growth could take place and bacterial reproduction could be restricted partly. Thus, we compared the mixotrophic growth and acetate conversion efficiency of an industrial oleaginous microalga [24] at different growth stages, taking into account both nitrogen and acetate concentrations. Finally, a novel sequential phototrophic–mixotrophic culture (SPMC) method, designed to enhance algal biomass production efficiency and inhibit bacterial growth, was used to cultivate *Graesiella* sp. WBG-1. We showed a successful scale-up of the SPMC in a 1000 m^2^ raceway pond, where an over 50% increase in biomass productivity was achieved.

## Methods

### Microalga and inoculum preparation

*Graesiella* sp. WBG-1, a green alga with large globose cells, was used in this study. It was provided by the Algae Culture Collection of Wuhan Botanical Garden, Chinese Academy of Sciences. This strain was originally isolated from Chenghai Lake, Yunnan province, China, with moderate lipid content of 17% DW and high protein content of 53% under nitrate-sufficient conditions. Our previous study showed that this fast-growing strain is an industrial strain capable of high lipid productivity in outdoor cultivation [24]. Also, *Graesiella* sp. WBG-1 showed mixotrophic growth when organic carbon (acetate) was available in a phototrophic culture.

The axenic colonies of *Graesiella* sp. WBG-1 were transferred from agar plates to liquid medium and grown at 25 °C in flasks shaken at 125 rpm under continuous illumination (100 μmol photons m^−2^ s^−1^). These cultures were used as seeds in the subsequent experiments.

The composition of the liquid medium used in this study [24] was as follows: 100 mg NaNO_3_, 24 mg KH_2_PO_4_, 75 mg MgSO_4_·7H_2_O, 36 mg CaCl_2_·2H_2_O, 6 mg Citric acid, 6 mg Fe-Ammonium citrate, 1 mg EDTA·Na_2_, 20 mg NaHCO_3_, 2.86 mg H_3_BO_3_, 1.8 mg MnCl_2_·4H_2_O, 0.22 mg ZnSO_4_·7H_2_O, 0.08 mg CuSO_4_·5H_2_O, 0.391 mg Na_2_MoO_4_·2H_2_O, and 0.0494 mg Co(NO_3_)_2_·6H_2_O per liter of deionized water. A concentrated solution of sodium acetate (1000×) was used to enrich the medium, as indicated in the text.

### Investigation of the effects of acetate on growth and lipid accumulation

The seed cultures were centrifuged, rinsed, and re-suspended in sterilized medium to an optical density of 0.1 at 540 nm. Then, aliquots (200 ml) of this cell suspension were transferred to closed column reactors (inner diameter 3 cm) for cultivation. The glass columns were maintained at 28 °C in a thermostatic water bath. Air enriched with CO_2_ (1% v/v CO_2_ in air) was passed through a 0.22 μm filter and then bubbled into the bottom of each column at a flow rate of 250 ml min^−1^. The columns were illuminated on one side at a light intensity of 300 μmol m^−2^ s^−1^ between 8:00 and 22:00 each day. The growth and lipid accumulation of *Graesiella* was studied under different initial acetate concentrations (0, 15, 29, 44, 59, 73, and 147 mM). Three replicate cultures were conducted in parallel.

### Laboratory investigation of the mixotrophic growth of *Graesiella* sp. WBG-1

A larger closed column reactor (inner diameter 8 cm, Additional file 1) was used in this experiment. Each column was inoculated with 400 ml newly prepared cell suspension (OD_540_ = 0.1). The columns were illuminated on one side at a light intensity of 300 μmol m^−2^ s^−1^ between 8:00 and 22:00 each day. Air enriched with CO_2_ (1% v/v CO_2_ in air) was passed through a sterile Millex syringe filter (0.22 μm) and then bubbled into the reactor at a flow rate of 250 ml min^−1^. The whole cultivation unit was placed in a thermostatic room to maintain the culture temperature at 28 °C.

Phototrophic cultivations were carried out first under the above conditions, to monitor changes in the residual nitrate concentration. Then, five mixotrophic culture strategies, namely E-FB, A-FB, P-FB, A-B, and P-B, were tested with supplementation of acetate to the phototrophic culture by batch/fed-batch mode before/after nitrate depletion. In the E-FB culture, 2.2 mM of acetate was fed to the reactor once a day for 8 days. In the A-FB culture, 2.2 mM of acetate was fed once a day at the beginning, 1st, and 2nd day, respectively. In the P-FB culture, 2.2 mM of acetate was fed once a day on the 4th, 5th, 6th, and 7th day, respectively. In addition, 6.6 mM of acetate was fed at the beginning of the A-B culture and 8.8 mM of acetate was fed on the 4th day of the P-B culture. A detailed description of the experimental setup is shown in Table 1. All of the cultivations were carried out in triplicate under axenic conditions.

**Table 1:** Experimental setup of the laboratory investigation using an 8 cm column reactor

	Phototrophy (CK)	Mixotrophy				
		E-FB	A-FB	P-FB	A-B	P-B
Culture period (days)	8	8	8	8	8	8
Acetate feed mode	None	Fed-batch	Fed-batch	Fed-batch	Batch	Batch
Feed at (days)	None	0, 1, …, 7	0, 1, 2	4, 5, 6, 7	0	4
Feed amount (mM day^−1^)	None	2.2	2.2	2.2	6.6	8.8
Culture volume (ml)	400	400	400	400	400	400

### Outdoor mixotrophic cultivation of *Graesiella* sp. WBG-1

Outdoor mixotrophic cultivations of *Graesiella* sp. WBG-1 were carried out at Chenghai, Yunnan province, China (N26°29′29.64″E100°40′56.12″). Two raceway ponds of different sizes were used in this study. The larger raceway pond was 65 m long and 16 m wide, giving an effective culture area of 1000 m^2^. The smaller pond had an effective culture area of 200 m^2^ (20 × 12 m). The structure and configuration of the 1000 m^2^ pond was similar to that of the 200 m^2^ pond as described in our previous study [24].

The raceway ponds were exposed to direct solar irradiation for at least 12 h for disinfection. Tap water was then used to fill the ponds to a depth of 20 cm, and sterile nutrient stock solutions were added to make the final medium. Finally, the seed cultures were inoculated, and the cell suspensions were ready for cultivation. The paddle wheels were set to a rotating speed of 20 rpm during the daytime by inverter control of motor speed. Culture pH was monitored using an automatic pH controller and maintained at the desired range of 9.0 ± 0.5 by dispersing pure CO_2_ to the culture broth. Natural solar irradiance, environmental temperature, and suspension temperature were monitored and logged in situ by an automatic weather station.

The cultures in the raceway ponds were first operated under phototrophic mode, then switched to mixotrophic mode once nitrate depletion was obtained. Acetate (2.2 mM) was added to the ponds every 24 h after nitrate depletion.

Two other 20 m^2^ raceway ponds covered by a greenhouse were used for high-quality seed preparation. Independent batch cultures in the 200 m^2^ pond and the 1000 m^2^ pond were carried out successively in the summers between 2014 and 2016.

### Analytical procedure

Cell growth was estimated by measuring the dry biomass concentration of the culture broth. About 10 ml of culture broth was filtered through a pre-dried GF/C glass microfiber filter paper (pore size 3 µm) and dried at 105 °C for 4 h, before being weighed to calculate the biomass dry weight (DW, g l^−1^).

The nitrate (NO_3_^−^), phosphate (PO_4_^3−^), and acetate (CH_3_COO^−^) concentrations in the medium were analyzed by ion chromatography [25]. The medium sample was diluted ten times with distilled water and filtered through a 0.45 μm filter membrane. This solution was directly injected into a Metrohm 940 Professional IC Vario instrument equipped with a chemical suppressor (MSM-A), a peristaltic pump unit, a conductivity detector, and a Metrosep A Supp 5-250/4.0 column. The mobile phase consisted of a mixture of Na_2_CO_3_ (3.2 mM) and NaHCO_3_ (1 mM) at a flow rate of 0.7 ml min^−1^. The volume of sample injection was 250 μl.

Algal cells were collected by centrifugation (5000 rpm for 5 min) and lyophilized (− 56 °C cryotrapping, 10–14 Pa vacuum) for biochemical analysis. For lipid quantification, 50 mg of dry algal biomass was fully grounded, transferred to a covered centrifuge tube, and then extracted with a mixture of *n*-hexane and ethyl acetate (1:1, v:v) at 50 °C for 20 min. The extraction was repeated three times, and all extracts were combined in a pre-weighed glass tube and then dried under nitrogen protection. The lipids were determined gravimetrically.

The fatty acid composition was determined by a direct transesterification method [26]. Briefly, 10 mg of the algal powder and 200 μl of chloroform/methanol (2:1, v/v) mixture were mixed in a 1.5 ml sample bottle, and then another 300 μl of methanol (containing 5% hydrochloric acid, v/v) was added before mixing well. The mixture was allowed to react at 85 °C for 1 h. After cooling to RT, 1 ml of *n*-hexane was added to extract the fatty acid methyl esters (FAMEs). The hexane layer was separated and dried with anhydrous sodium sulfate. FAMEs were analyzed by gas chromatography (Agilent 7890A) using an HP-5 Phenyl Methyl Siloxan column (30 m × 0.32 mm × 0.25 µm) and a flame ionization detector. Then, 1 µl of fatty acid methyl esters solution was injected with a splitting ratio of 5:1. The heating program was as follows: 150 °C for 2 min, then increase to 250 °C at a rate of 10 °C per min, and hold for 8 min. A standard FAME Mix (Sigma-Aldrich) was used for fatty acid identification.

About 2 mg of the lyophilized algal powder was used for total carbon (TC) quantification [27]. The algal powder was packaged in a tin capsule and combusted at 950 °C using a Vario TOC select instrument running in solid mode. The carbon in the biomass was converted to CO_2_ after combustion and quantitatively determined using an NDIR detector.

Bacterial cell numbers in the outdoor mixotrophic cultivations were estimated by colony-forming unit (CFU) counting [28]. The algal suspensions were streaked on lysogeny broth (LB) agar plates after three gradient dilutions. These plates were then incubated at 37 °C for 24–48 h. The CFUs were manually counted and only reads between 30 and 300 CFU were further analyzed.

### Calculations

Volumetric biomass concentration (*C*_biomass_, g l^−1^) was equal to the biomass dry weight (DW). Areal biomass concentration (*C*_biomass_, g m^−2^) was calculated by Eq. (1):

1 $$C_{\text{biomass}} = \frac{DW \times V}{S},$$

with DW as biomass dry weight (g l^−1^), *V* as culture volume (l), and *S* as illuminated area (m^2^).

Biomass productivity at any time point *t* (*P*_biomass_ (*t*), g m^−2^ day^−1^) was calculated according to Eq. (2):

2 $$P_{\text{biomass}} \left( t \right) = \frac{{C_{\text{biomass}} \left( t \right) - C_{\text{biomass}} \left( 0 \right)}}{t},$$

with *t* as cultivation time (days) and *C*_biomass_ (*t*) as areal biomass concentrations (g m^−2^) at cultivation time *t* (day).

Lipid productivity at any time point *t* (*P*_lipid_ (*t*), g m^−2^ day^−1^) was calculated according to Eq. (3):

3 $$P_{\text{lipid}} \left( t \right) = \frac{{C_{\text{biomass}} \left( t \right) \times C_{\text{lipid}} \left( t \right) - C_{\text{biomass}} \left( 0 \right) \times C_{\text{lipid}} \left( 0 \right)}}{t},$$

with *t* as cultivation time (days) and *C*_lipid_ (*t*) as lipid content (% DW) at cultivation time *t* (day).

Average daily light intensity (*I*_av_, mol m^−2^ day^−1^) was calculated according to Eq. (4):

4 $$I_{\text{av}} = \frac{{\mathop \sum \nolimits_{0}^{t} I_{\text{inc}} }}{t},$$

with *I*_inc_ as daily incident light intensity (mol m^−2^ s^−1^) measured by the automatic weather station.

Specific growth rate (µ, day^−1^) was calculated according to Eq. (5):

5 $$\mu = {\raise0.7ex\hbox{${\ln \left( {C_{\text{biomass}} \left( t \right)/C_{\text{biomass}} \left( 0 \right)} \right)}$} \!\mathord{\left/ {\vphantom {{\ln \left( {C_{\text{biomass}} \left( t \right)/C_{\text{biomass}} \left( 0 \right)} \right)} t}}\right.\kern-0pt} \!\lower0.7ex\hbox{$t$}}.$$

Acetate conversion efficiency (ACE, g g^−1^) was calculated according to Eq. (6):

6 $${\text{ACE}} = {\raise0.7ex\hbox{${\Delta C_{{{\text{biomass,}} M}} - \Delta C_{{{\text{biomass,}} P}} }$} \!\mathord{\left/ {\vphantom {{\Delta C_{{{\text{biomass,}} M}} - \Delta C_{{{\text{biomass,}} P}} } {M_{\text{acetate}} }}}\right.\kern-0pt} \!\lower0.7ex\hbox{${M_{\text{acetate}} }$}},$$

with Δ*C*_biomass, M_ and Δ*C*_biomass, P_ as the net increase of biomass concentrations of the mixotrophic and phototrophic culture, respectively, and *M*_acetate_ as the amount of Na-acetate (g) supplemented.

All of the results were analyzed for variance with SAS 9.0 at a significance level *α* = 0.05. Tukey’s multiple comparison tests were done where applicable.

## Results

### Effects of acetate on *Graesiella* sp. WBG-1 growth and lipid accumulation

**Fig. 1:**
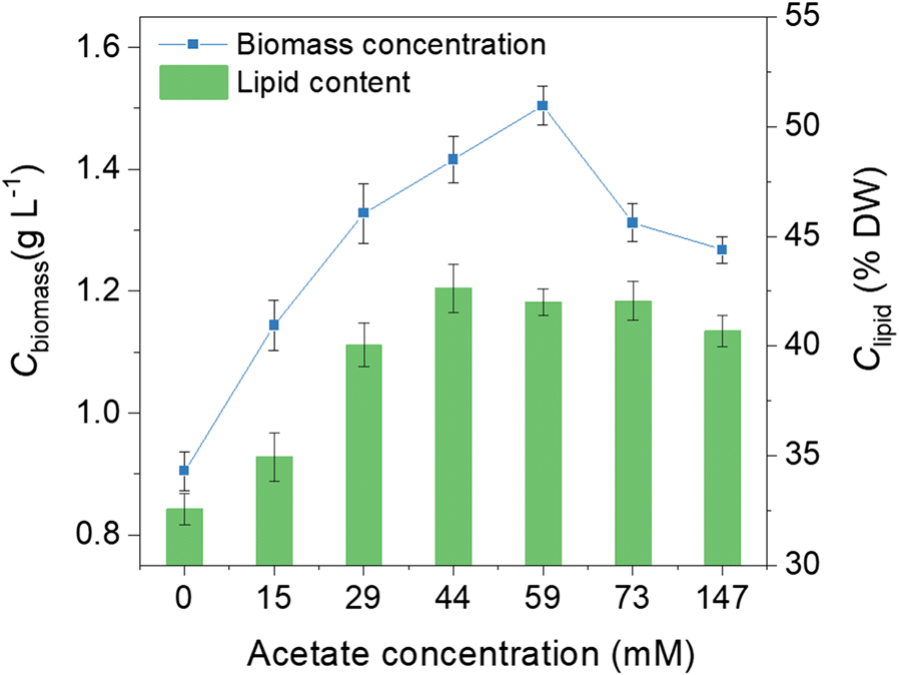
Effects of acetate supplementation on cell growth (*C*_biomass_, blue line) and lipid accumulation (*C*_lipid_, green column) of *Graesiella* sp. WBG-1

Batch cultivations were carried out in column reactors (inner diameter 3 cm) to compare the growth and lipid accumulation of *Graesiella* sp. WBG-1 between mixotrophic cultures (15, 29, 44, 59, 73, and 147 mM acetate) and phototrophic culture (0 mM acetate). Enhanced cell growth was observed in mixotrophic cultures in which the concentrations of acetate were below 59 mM. As shown in Fig. 1, the biomass concentration (*C*_biomass_) of *Graesiella* sp. WBG-1 increased with increase in acetate concentration from 15 mM to 59 mM. The highest *C*_biomass_ was about 1.5 g l^−1^, which was 1.7-times as high as that of the phototrophic culture. Further increases in acetate concentration inhibited the growth of *Graesiella* sp. WBG-1 and the *C*_biomass_ decreased sharply to 1.2 g l^−1^.

The highest lipid content (*C*_lipid_) of 42.7% DW was achieved with an acetate concentration of 44 mM (Fig. 1). Further increases in acetate concentration had little influence on the lipid accumulation of the alga. The *C*_lipid_ only slight decreased (40.7% DW) under the highest acetate concentration of 147 mM. Overall, the *C*_biomass_ and *C*_lipid_ of the mixotrophic cultures were both significantly higher than that of the phototrophic culture. These results confirmed the ability of *Graesiella* sp. WBG-1 to utilize acetate under tolerable acetate concentrations. Though the lipid content of the mixotrophic culture was significantly higher than that of the phototrophic culture, more data are needed to identify the direct effects of acetate on lipid accumulation, since the culture was nitrogen limited (the initial nitrate concentration was 1.18 mM) and nitrogen limitation was the most effective factor to induce TAG synthesis [9].

### Acetate consumption under different acetate feeding strategies

A preparative experiment was carried out in the closed column reactor (inner diameter 8 cm) to investigate the changes of residual nitrate concentrations in phototrophic and mixotrophic cultivations of the alga *Graesiella* sp. WBG-1. It was suggested by the pre-experiment that the nitrate concentration of the phototrophic culture dropped below 0.01 mM on the 3rd day, which was considered to be nitrate-depleted. In the mixotrophic culture, nitrate was consumed faster and was exhausted by the 2nd day. Therefore, the phototrophic and mixotrophic cultures were nitrate sufficient before the 3rd day and 2nd day, respectively. Thereafter, the two cultures were both nitrate depleted.

**Fig. 2:**
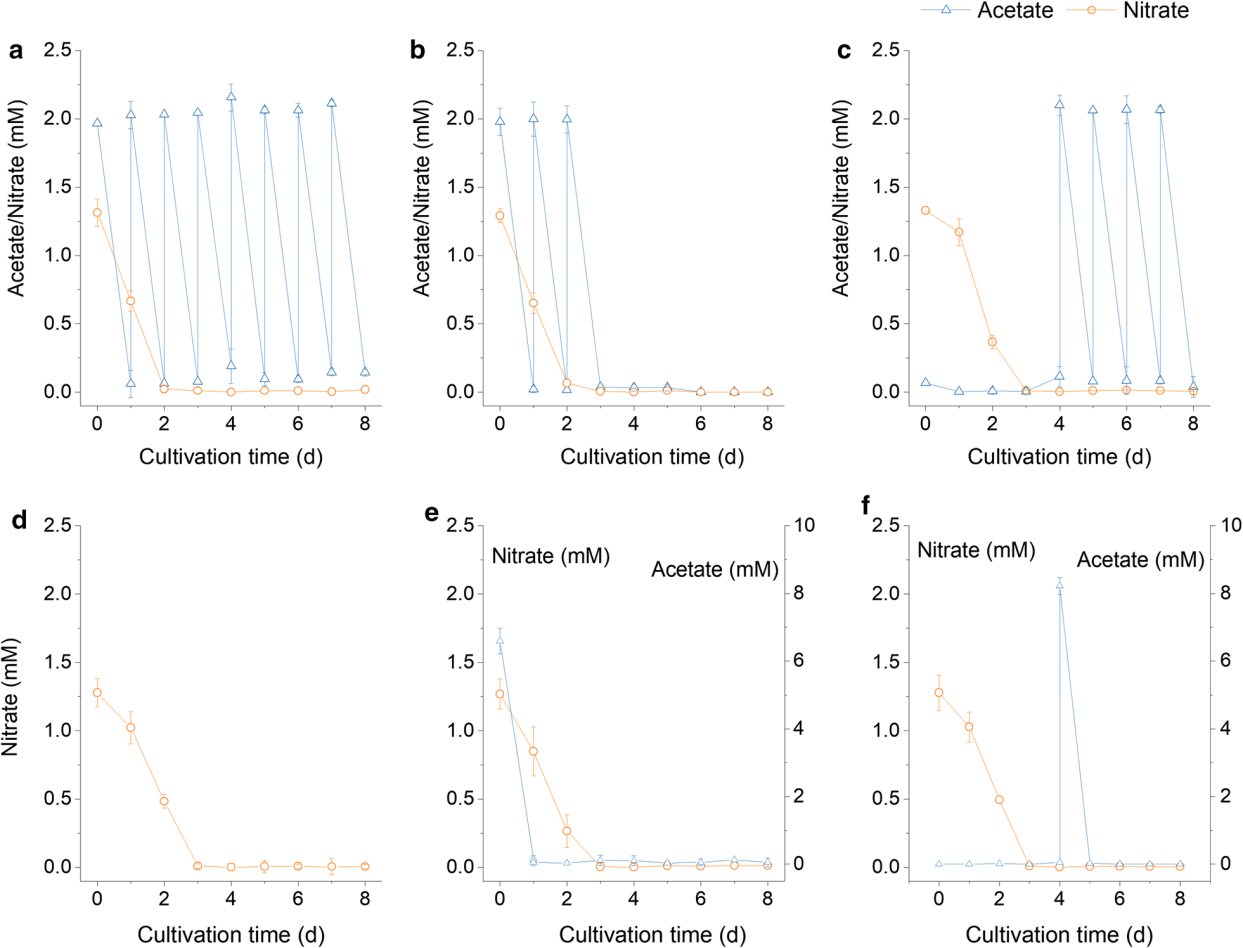
Changes of nitrate (orange circle) and acetate (blue up triangle) concentrations under different acetate feeding strategies. The *Graesiella* cultures were supplied with the same amount of nitrate (~ 1.3 mM) at the beginning, and acetate sodium were supplemented either by fed-batch mode during the whole culture period (E-FB strategy, **a**), before nitrate depletion (A-FB strategy, **b**) and after nitrate depletion (P-FB strategy, **c**), or by batch mode before nitrate depletion (A-B strategy, **e**) and after nitrate depletion (P-B strategy, **f**). The phototrophic culture (**d**) was not supplemented with any organic carbon during cultivation

According to the patterns of nitrate consumption, different acetate feeding strategies (Table 1) were applied to the *Graesiella* sp. WBG-1 cultures in the closed 8 cm column reactor. The measured values of acetate and nitrate concentrations are shown in Fig. 2. The patterns of nitrate consumption were in line with that in the pre-experiment. All of the acetate fed in the experiment was consumed completely (detection limit 1 μg l^−1^) and the observed maximum rate of consumption was 8.23 mmol l^−1^ day^−1^ in this study.

### Comparison of mixotrophic growth under different acetate feeding strategies

**Fig. 3:**
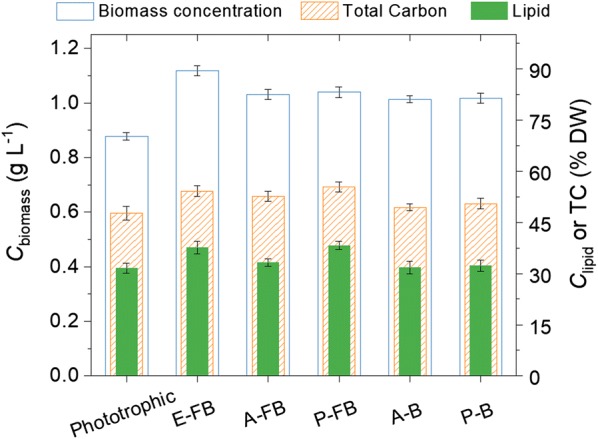
Comparisons of biomass concentration (*C*_biomass_, blue open column), biomass carbon (TC, orange column filled with slash line), and lipid content (*C*_lipid_, green filled column) under different acetate feeding strategies (E-FB, A-FB, P-FB, A-B, P-B)

Different *C*_biomass,_ but similar *C*_lipid_ of *Graesiella* sp. WBG-1 were observed between the five acetate feeding strategies after 8 days of cultivation (Fig. 3). Notably, the highest *C*_biomass_ (1.12 g l^−1^) was obtained in the E-FB culture, which was fed with 2.2 mM of acetate every day throughout cultivation (Fig. 2a). This represents a 29% increase over the simple phototrophic culture. Moreover, *C*_lipid_ of the E-FB culture reached a value of 37.6% DW, which was significantly higher than that of the phototrophic culture. When nitrate was added in fed-batch mode before (A-FB, Fig. 2b) or after (P-FB, Fig. 2c) nitrate depletion, a fairly small increase in *C*_biomass_ (1.06 g l^−1^ and 1.01 g l^−1^, respectively) was observed compared to the phototrophic culture. At the same time, the P-FB culture reached the highest *C*_lipid_ of 38.7% DW, which was significantly higher than that of the phototrophic culture. In contrast, the *C*_lipid_ of the A-FB culture was only 33.2% DW, which was almost the same as that of the phototrophic culture. The A-B and P-B cultures, to which nitrate was added in batch mode before (A-B, Fig. 2e) or after (P-B, Fig. 2f) nitrate depletion, showed consistent results. Overall, in comparison to the phototrophic culture, an increase of about 15–29% in *C*_biomass_ was achieved in the five mixotrophic cultures, where the highest *C*_lipid_ were achieved in the E-FB and P-B cultures.

Elemental analysis showed that the total carbon content (TC) of the algal biomass in E-FB, A-FB, and P-FB cultures were significantly higher than that of the phototrophic culture (Fig. 3). The highest TC (55.3% DW) was achieved in the P-FB culture. Nevertheless, no significant differences were observed between the batch-operated mixotrophic cultures (A-B, P-B) and the phototrophic culture, even though slightly higher TC of 49.4% DW and 50.5% DW were detected in the A-B and P-B cultures, respectively. In fact, TC content is correlated with the amount of storage compounds (such as lipid/TAGs).

The fatty acid profiles of *Graesiella* sp. WBG-1 under different acetate feeding strategies are shown in Table 2 and Additional file 1. In contrast to the variations in biomass and lipid content, minor differences were observed in fatty acid composition between the five mixotrophic cultures. However, the differences between the mixotrophic and phototrophic cultures were still noteworthy. C16 and C18 fatty acids were the most abundant components in both mixotrophic and phototrophic cultures, with higher amounts of saturated and monounsaturated fatty acids, such as palmitic acid (C16:0), stearic acid (C18:0), palmitoleic acid (C16:1), and oleic acid (C18:1) observed in the mixotrophic cultures. Nevertheless, the amount of polyunsaturated fatty acid, such as linoleic acid (C18:2) and α-linolenic acid (C18:3), was lower in the mixotrophic than the phototrophic cultures. These results are consistent with previously reported findings [29, 30].

**Table 2:** Components and relative abundance of fatty acids under different culture strategies using the 8 cm column reactor

Fatty acids	Relative abundance (%)					
	Phototrophic	E-FB	A-FB	P-FB	A-B	P-B
C14:0	0.59	0.73	0.69	0.81	0.75	0.67
C16:0	27.26	28.18	28.11	29.12	28.72	28.46
C18:0	2.87	3.07	3.15	3.00	3.31	3.58
C20:0	0.43	0.42	0.55	0.45	0.40	0.35
C22:0	0.09	0.07	0.07	0.06	0.08	0.10
C24:0	0.04	0.04	0.05	0.04	0.03	0.02
C14:1	0.08	0.05	0.06	0.10	0.07	0.10
C16:1	2.44	3.15	3.32	3.22	4.01	3.37
C18:1	37.12	38.76	38.79	38.74	38.89	40.02
C20:1	1.18	1.07	1.12	1.32	1.17	1.03
C16:2	7.06	6.81	7.01	7.23	6.87	7.14
C18:2	9.09	8.90	8.77	7.59	7.41	7.57
C18:3	11.42	8.57	8.10	8.14	8.24	7.39
C20:5	0.33	0.18	0.21	0.18	0.05	0.20

### Sequential phototrophic–mixotrophic cultivation (SPMC) of *Graesiella* sp. WBG-1 in large raceway pond

**Fig. 4:**
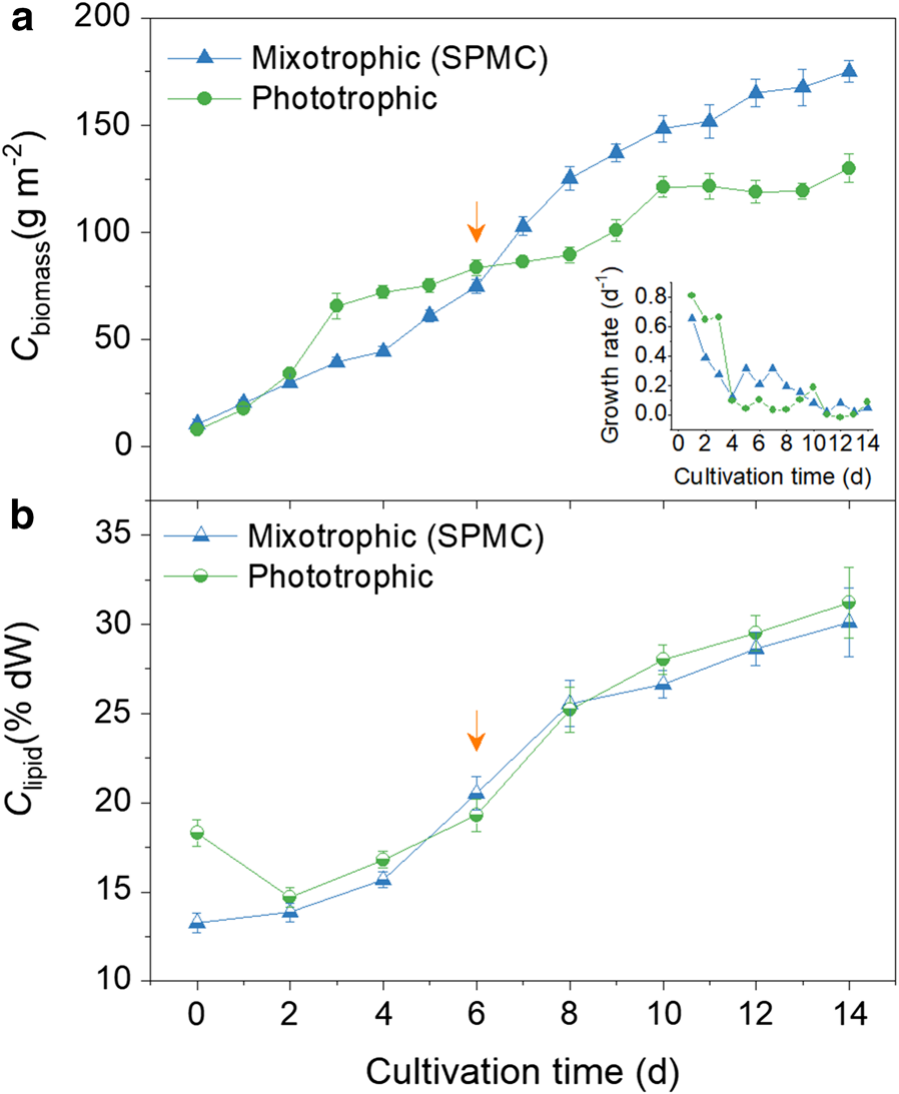
Growth (*C*_biomass_, **a**) and lipid accumulation (*C*_lipid_, **b**) of *Graesiella* sp. WBG-1 in a 200 m^2^ raceway pond. The mixotrophic cultivations (blue up triangle) were operated by SPMC method in May 14–28/2015. Down arrows indicate the first supplementation of acetate. Data of the phototrophic cultivation (green circle) are cited from our earlier study [24]. Error bars show the standard deviations of at least three measurements

The laboratory investigations suggested the existence of a window period for acetate supplementation during the phototrophic culture of *Graesiella* sp. WBG-1. During this window period, algal growth could be enhanced by mixotrophic growth under nitrate-depleted conditions (Figs. 2, 3). The P-FB culture strategy, which had such a window period and resulted in higher *C*_lipid_ compared with the P-B culture, was therefore tested in a 200 m^2^ raceway pond to evaluate the algal production efficiency as well as the bacterial growth of this new cultivation method under outdoor conditions. We refer to this strategy as the sequential phototrophic–mixotrophic cultivation (SPMC). A total of six fed-batch cultivations (SPMC) of *Graesiella* sp. WBG-1 were carried out in a 200 m^2^ raceway pond in the years 2014 and 2015 (Table 3, Fig. 4, Additional file 2). The cultivations were mainly conducted in May, June, July, and September, because of the warm sunny climate with little rainfall at the test site during these months.

**Table 3:** Overview of the SPMCs conducted in open raceway pond

Cultivations	Culture scale (m^2^)	*I*_av_ (µmol m^−2^day^−1^)	*P*_biomass_ (g m^−2^day^−1^)	*C*_lipid_ % DW	*P*_lipid_ (g m^−2^day^−1^)	Increment in *P*_biomass_ (%)	Refs.
Phototrophic (Jun. 2013)	200	45	8.7	31.8	2.9	0	[24]
Phototrophic (Jul. 2013)	200	36	7.2	33.4	2.5		
Phototrophic (May 2013)	200	39	6.2	29.4	2.0		
SPMC (Jun. 2014)	200	26	9.6	34.1	3.4	43.9	This study
SPMC (Jul. 2014)	200	29	11.7	30.1	3.7		
SPMC (Sep. 2014)	200	30	10.5	35.4	3.8		
SPMC (May 2015)	200	44	11.8	30.1	3.7	57.0	This study
SPMC (Jun. 2015 A)	200	52	12.4	33.8	4.2		
SPMC (Jun. 2015 B)	200	37	10.5	32.7	3.4		
SPMC (Jul. 2016)	1000	42	12.5	31.6	4.1	56.6	This study
SPMC (Sep. 2016 A)	1000	39	11.8	34.2	4.1		
SPMC (Sep. 2016 B)	1000	36	10.3	30.1	3.2

The microalga *Graesiella* sp. WBG-1 were grown phototrophically from the beginning to the 6th day of SPMC. The *C*_biomass_ increased linearly with cultivation time and nitrate was gradually exhausted from the medium during this period. The culture was then nitrate depleted and the microalgal growth rate decreased to some extent. Taking the SPMC conducted in May 14–28/2015 as an example (Fig. 4), the average growth rates of SPMC were 0.33 day^−1^ before nitrate depletion, which was similar to the phototrophic culture (0.39 day^−1^) in our previous study [24]. Although the growth rate decreased to 0.11 day^−1^ during the rest cultivation time of the SPMC, it was twice as high as that of the phototrophic culture. As a result, the *C*_biomass_ at the end of SPMC was 175.2 g m^−2^, which was 1.4 times as high as that of the phototrophic culture. This suggests that the supplementation of acetate significantly enhanced the growth of *Graesiella* sp. WBG-1 in the raceway pond.

The *C*_lipid_ in the SPMC (Fig. 4) changed similarly to that in the phototrophic culture [24]. The *C*_lipid_ on the 6th day was about 20% DW for both cultures. It increased to 30.1% DW after 8 days of mixotrophic cultivation under nitrate-depleted conditions (SPMC). Meanwhile, the *C*_lipid_ of the phototrophic culture was 31.2% DW at the same time, which was a non-significant increase compared to the SPMC.

**Fig. 5:**
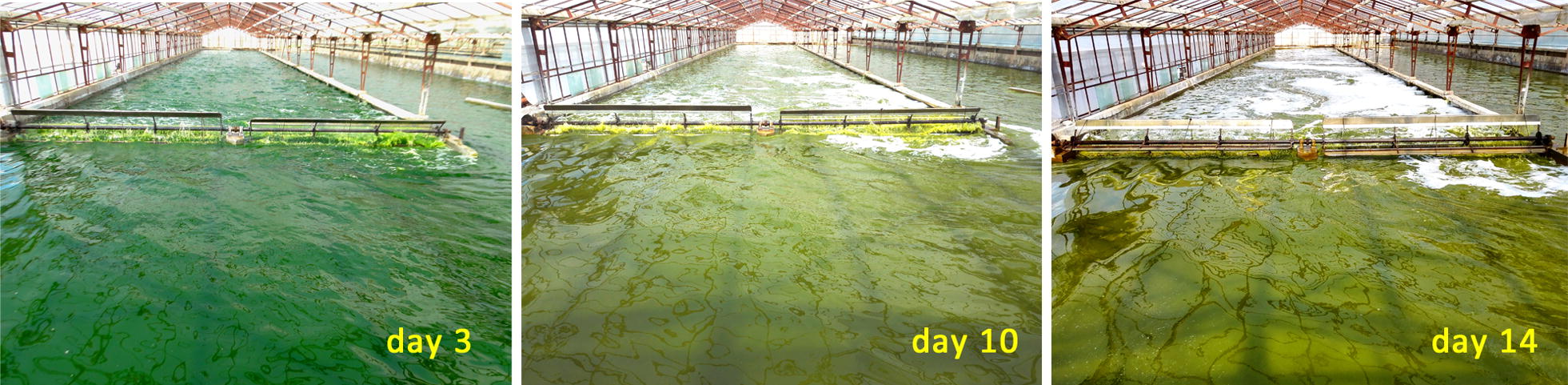
Color evolution of algal suspension in the 1000 m^2^ raceway pond operated under the SPMC regime

**Fig. 6:**
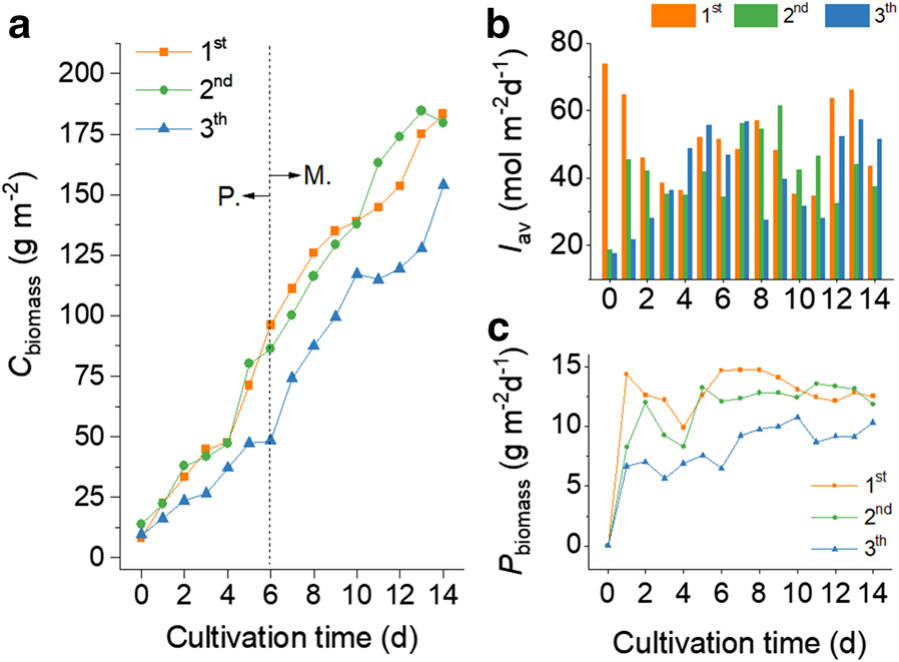
Time evolutions of biomass concentration (*C*_biomass_, **a**), biomass productivity (*P*_biomass_, **c**) and average light intensity (*I*_av_, **b**) during SPMC in the 1000 m^2^ raceway pond. Colors indicate the different SPMCs on Jul. 26–Aug. 9/2016 (1st), Sep. 2–16/2016 (2nd) and Sep. 20–Oct. 4/2016 (3th), respectively. Dashed line shows switching of the culture from phototrophic regime to mixotrophic regime

Applications of the SPMC strategy in a commercial open pond with a culture area of 1000 m^2^ were conducted in the year 2016. The *Graesiella* sp. WBG-1 suspensions changed gradually from a dark green color to yellow-green during the cultivations (Fig. 5), suggesting the degradation of chlorophylls under nitrate deficiency [9]. Yields of the three SPMCs were comparable to those obtained in the 200 m^2^ raceway pond. For example, a sustained increase in *C*_biomass_ was observed throughout the 14 days of the first 1000 m^2^ SPMC test conducted between July 26 and August 9, 2016 (Fig. 6), even though the algal cells were under nitrate-depleted condition after the 6th day. A *C*_biomass_ of 96.1 g m^−2^ was obtained on the 6th day, and the highest *C*_biomass_ (183.3 g m^−2^) achieved at the end, that is on the 14th day, was almost 23 times as high as the *C*_biomass_ at the beginning of the cultivation. Solar intensity during the cultivation ranged from 34.6 to 73.8 mol m^−2^ day^−1^. *P*_biomass_ varied according to varying intensities of solar irradiation (Fig. 6). An average *P*_biomass_ of 12.5 g m^−2^ day^−1^ over the 14-day cultivation was obtained, with the highest *P*_biomass_ of 14.7 g m^−2^ day^−1^ achieved on the 8th day under 57 mol m^−2^ day^−1^ of solar irradiation.

**Fig. 7:**
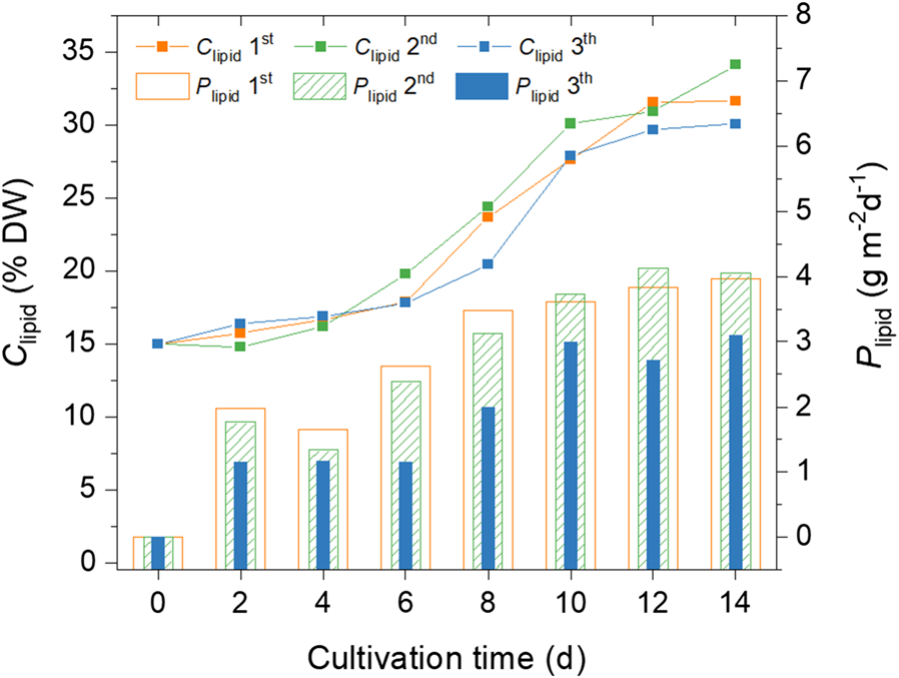
Time evolutions of lipid content (*C*_lipid_, line) and lipid productivity (*P*_lipid_, column) during SPMC in the 1000 m^2^ raceway pond. Colors indicate the different SPMCs on Jul. 26–Aug. 9/2016 (1st), Sep. 2–16/2016 (2nd) and Sep. 20–Oct. 4/2016 (3th), respectively

Lipid accumulation mainly occurred in the mixotrophic stage of the 1000 m^2^ SPMC when nitrate was depleted from the medium (Fig. 7). For example, the *C*_lipid_ was 17.9% DW on the 6th day of the first 1000 m^2^ SPMC test, only a small increase compared to that at the beginning (15.1% DW). However, the *C*_lipid_ increased to 31.6% DW in the remaining 8 days of cultivation. As a result, the daily *P*_lipid_ increased from 1.98 g m^−2^ day^−1^ on the 2nd day to 3.96 g m^−2^ day^−1^ on the 14th day of the first 1000 m^2^ SPMC.

### Evaluations of bacterial contamination during SPMC in the 1000 m^2^ raceway pond

**Fig. 8:**
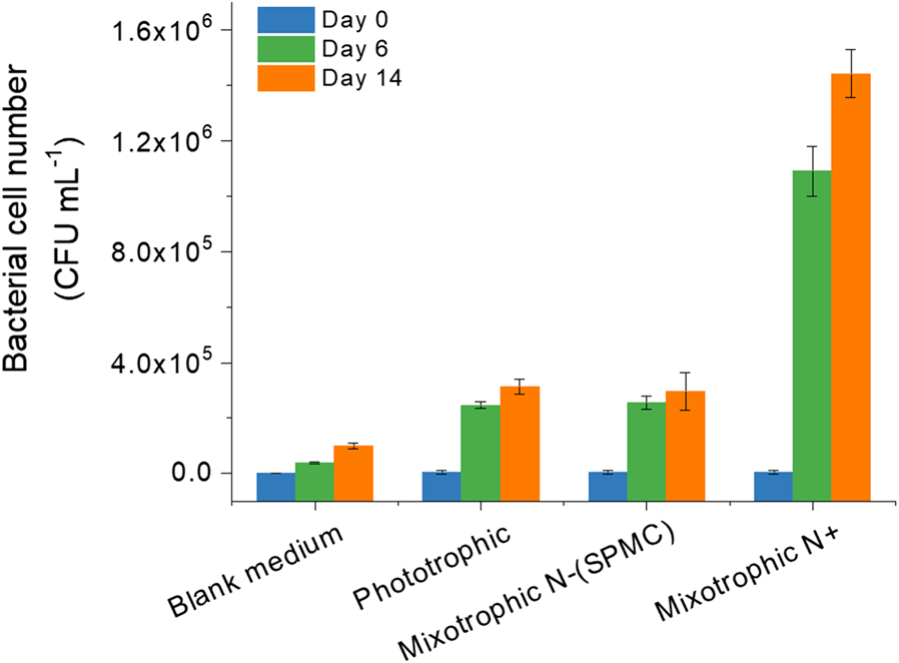
Bacterial cell densities during the outdoor cultivation of *Graesiella* sp. WBG-1

The SPMC culture broth was subjected to CFU estimations to quantify the bacterial cell densities (Fig. 8). The bacterial cell numbers at the beginning of SPMC and phototrophic cultivations were both about 7 × 10^3^ CFU ml^−1^, where the blank medium (negative control) was 9 × 10^2^ CFU ml^−1^. After the first 6 days of cultivation, the bacterial cell numbers in the SPMC and the phototrophic culture were 2.56 × 10^5^ CFU ml^−1^ and 2.47 × 10^5^ CFU ml^−1^, respectively. Most importantly, the bacterial cell number only increased to 2.97 × 10^5^ CFU ml^−1^ after the later 8 days of mixotrophic cultivation in the SPMC, which was similar to that of the phototrophic culture (3.13 × 10^5^ CFU ml^−1^). However, the bacterial cell number was found to increase sharply to 1.1 × 10^6^ CFU ml^−1^ on the 6th day and 1.4 × 10^6^ CFU ml^−1^ on the 14th day in another comparative cultivation where acetate was added before the 6th day, when there was sufficient nitrate in the medium. As such, the SPMC strategy inhibited bacterial growth to a certain extent.

## Discussion

### Growth rates and acetate conversion efficiencies during different growth stages of *Graesiella* sp. WBG-1

Acetate is a kind of organic carbon that can be easily utilized by many microalgae [17]. It is reported that acetate is assimilated by the glyoxylate cycle and utilized for carbon assimilation via gluconeogenesis, or by the TCA cycle to sustain respiration and mitochondrial ATP production [31]. In this study, the growth rates and acetate conversion efficiencies of *Graesiella* sp. WBG-1 were compared under five acetate feeding strategies with varying acetate and nitrate availability, particularly under nitrate-depleted conditions.

Our results show differences in the biomass concentration (*C*_biomass_) of *Graesiella* sp. WBG-1 between the five acetate feeding strategies (Fig. 3). Mixotrophy increased the *C*_biomass_ by more than 15% in contrast to the phototrophic culture, regardless of how and when acetate was added. The effects of acetate supplementation at different culture stages (with regards to nitrate availability) on algal growth rate (μ) were varied (Table 4). Owning to mixotrophy, the growth rates reached 1.96, 1.94, and 1.93 day^−1^ in the E-FB, A-FB, and A-B cultures before nitrate depletion, respectively, which were more than 1.5 times as high as that of the phototrophic culture (1.23 day^−1^). Nitrate depletion decreased the growth rates by over 90% in all cultures. However, the supplementation of acetate under nitrate-depleted conditions gave rise to enhanced cell growth. For example, the growth rates of the P-FB and P-B cultures after nitrate depletion were 0.12 and 0.11 day^−1^, respectively, which were significantly higher than the growth rate of the phototrophic culture (0.08 day^−1^).

**Table 4:** Growth rate and acetate conversion efficiency at different culture stage in the 8 cm column reactor

Cultures	Before nitrate depletion			After nitrate depletion		
	Δ*C*_biomass_ g l^−1^	μ day^−1^	ACE g g^−1^	Δ*C*_biomass_ g l^−1^	μ day^−1^	ACE g g^−1^
Phototrophic	0.56	1.23	–	0.31	0.08	–
E-FB	0.70	1.96	0.26	0.41	0.08	0.11
A-FB	0.67	1.94	0.21	0.35	0.07	–
P-FB	0.57	1.24	–	0.46	0.12	0.21
A-B	0.65	1.93	0.17	0.35	0.07	–
P-B	0.58	1.25	–	0.43	0.11	0.16

Furthermore, the E-FB and P-FB cultures showed the highest biomass carbon content and lipid content among the five mixotrophic cultures. These results suggest that the supplementation of acetate by fed-batch mode has an advantage over the supplementation of acetate by batch mode. This is further supported by the observation that acetate conversion efficiencies (ACEs) of *Graesiella* sp. WBG-1 in the E-FB, A-FB, and P-FB cultures were higher than those in the A-B and P-B cultures (Table 4). In detail, up to 0.17 g of biomass was produced for every 1 g of Na-acetate supplemented by batch mode. However, the ACEs increased up to 0.26 g g^−1^ if acetate was supplemented by fed-batch mode. Particularly in the P-FB culture, supplementation of acetate by fed-batch mode under nitrate-depleted conditions resulted in a relatively high ACE of 0.21 g biomass g^−1^ acetate, highlighting the efficient utilization of acetate by *Graesiella* sp. WBG-1. Nevertheless, the high growth rate before nitrate depletion of the E-FB culture, and therefore the high consumption of the intracellular N storage pool [32], which will be discussed below, resulted in low light availability [33] and excessive nitrate limitation, and finally made a low ACE after nitrate depletion.

The above results clearly demonstrate the existence of two window periods during the phototrophic cultivation for growing alga mixotrophically. The first window period is found at the early stage of the culture, when nitrate and other nutrition are sufficient in the medium. Supplementation of acetate during this period to form a mixotrophic culture is common, but may result in serve bacterial contamination under open culture conditions [34, 35]. In this study, we focused on the second window period, that is, when nitrate is depleted from the medium. The aim was to identify how the microalga are able to grow mixotrophically under nitrate-depleted conditions but the bacteria reproduction is limited. The latter will be discussed in the next subsection as the laboratory experiments were conducted under axenic conditions.

Many green algae have the ability to luxury uptake of extracellular nitrogen and phosphorus under favorable conditions [36–38], showing that algal cells can internalize nutrition (such as nitrate) much faster than its effective use for growth. The microalga *Graesiella* sp. WBG-1 is able to absorb up to 5.88 mM of nitrate in a very short time (i.e., 3 days) in a column PBR [24]. So, little nitrate was available in the culture medium during the majority of the cultivation time in this study (Additional file 3). Some of the assimilated nitrate-nitrogen was reduced and immediately incorporated to carbon skeletons by rendering glutamate [39], while the rest served as an intracellular N storage pool in form of amino acids, amino compounds, nitrate, ammonium, protein, and chlorophyll [32]. These N reserves were used for algal growth for several days after the depletion of external nitrogen. This growth phase, which we regard as the second window period for acetate supplementation, can last for 5–10 days, depending on the size of the N storage pool and other environmental conditions, and is key to establishing a mixotrophic algae culture in open reactors.

### Enhancing algal biomass production in open raceway pond by SPMC

According to the laboratory investigation, we carried out an outdoor mixotrophic cultivation of *Graesiella* sp. WBG-1 in a 200 m^2^ raceway pond. The alga first grew phototrophically, then acetate was supplemented by fed-batch mode during the second window period once nitrate were depleted from the medium. The cultivation, which we denoted as sequential phototrophic–mixotrophic cultivation (SPMC), showed enhanced *C*_biomass_ and *P*_biomass_, but similar *C*_lipid_ (Fig. 4) in comparison to the phototrophic cultivation of the microalga *Graesiella* sp. WBG-1 reported before [24]. The changes in *C*_biomass_ in the 200 m^2^ SPMCs, which were in form of linear increases throughout the cultivation, were different from the slowdown of *C*_biomass_ after nitrate depletion in the phototrophic cultivation. Overall, a 43–57% increase in the *P*_biomass_ of *Graesiella* sp. WBG-1 was achieved in the SPMCs (200 m^2^ raceway pond) conducted in the year 2014 and 2015.

Further tests of the SPMC method in a larger commercial raceway pond (1000 m^2^ culture area) obtained comparable *P*_biomass_ and *C*_lipid_ to those in the 200 m^2^ SPMCs. Sustained increases in *C*_biomass_ of the three 1000 m^2^ SPMCs lead to average daily *P*_biomass_ of 12.5, 11.8, and 10.3 g m^−2^ day^−1^. At the same time, the *C*_lipid_, the majority of which is TAGs (Additional file 4), reached 31.6, 34.2, and 30.1% DW, respectively (Table 3). In contrast to the phototrophic cultivation of *Graesiella* sp. WBG-1 [24], the *P*_biomass_ of the 1000 m^2^ SPMCs increased by 56.6%, suggesting that the second window period can be used to form a mixotrophic algal culture. Moreover, this confirms that SPMC is an effective method for enhancing oleaginous algal biomass production in open raceway ponds.

As we previously reported, the accomplishment of a lipid content higher than 30% DW in outdoor cultivation of oleaginous green algae is of great importance, not only because of the marked accumulation of TAGs in microalgal cells, but also due to the general trend that the yield of microalgae declines with increasing culture volumes [24]. Our previous study showed an average *P*_biomass_ of 7.4 g m^−2^ day^−1^ with an average *C*_lipid_ of 31.5% DW in the phototrophic cultivation of the microalga *Graesiella* sp. WBG-1 in a 200 m^2^ raceway pond [24]. The levels of solar irradiation during the three 1000 m^2^ SPMCs in this study were close to those of the phototrophic cultivations. Yet, mass transfer (roughly liquid mixing) in the former was lower than that of the latter, owing to the increased culture area (1000 m^2^) and decreased liquid flow speed. However, comparable *C*_lipid_ were achieved in the two cultivations. What is more important, significantly enhanced *P*_biomass_ and thus enhanced *P*_lipid_ were achieved in the 1000 m^2^ raceway pond using the SPMC method (Table 3). These results demonstrate the effectiveness of SPMC in enhancing oleaginous algal biomass production on a commercial scale. Furthermore, our results also show that more than 90% of the oil derivates are C16 and C18 FAMEs (Table 2), which are quite suitable for producing biodiesel [5].

Evidently, some of the increases in *P*_biomass_ (Table 3) were due to improved culture conditions, such as solar irradiation and temperature. The contributions of these factors are difficult to remove quantitatively from *P*_biomass_ because the SPMCs and the phototrophic cultivations were conducted at different times. But considering the correlations between *I*_av_ and *P*_biomass_ in the phototrophic cultivation of the microalgae *Graesiella* sp. WBG-1 in open raceway ponds (Additional file 5), the contribution of every 10 mol of improved irradiation (photons m^−2^ day^−1^) on *P*_biomass_ should not be larger than 2.6 g DW m^−2^ day^−1^. As such, it is reasonable to suggest that SPMC increased the *P*_biomass_ of *Graesiella* sp. WBG-1 by at least 50%.

### Application prospect of the SPMC technique in commercial algal biomass production

One of the most important problems associated with mixotrophic cultivation of microalgae in an open system is bacterial contamination [16], which has negative effects on algal growth [40] and on the quality of the microalgal product. Generally, bacterial contamination is unavoidable in outdoor microalgae cultivation [41] and is intensified if organic carbon is available in the medium. For example, the relative bacterial abundance of a *Chlorella* seed culture was found to be approximately 10^4^ CFU ml^−1^, which increased to 1.4 × 10^7^ CFU ml^−1^ after 5 days of mixotrophic incubation [42]. However, results of the CFU estimation in this study suggest that bacterial populations in the SPMC are limited to a small size comparable to that of the phototrophic cultivations (Fig. 8). It has been reported that the number of bacteria in aquatic environments is positively correlated with the concentrations of phosphorus and nitrogen [43, 44]. Unlike the microalgae, which have shown the ability to internalize nitrate much faster than its effective use for growth, the bacterial growth is more accurately described as a function of the nutrients readily available in the culture media [16]. Thus, nitrate and phosphorus deficiency during the mixotrophic stage of SPMC (Additional file 3) explains why bacterial growth was inhibited. This finding is further supported by the observation that the medium at the end of SPMC was always clear and not milk-white or turbid, the latter of which indicates serious contamination by bacteria. Moreover, the supplementation of acetate before nitrogen and phosphorus depletion increased the abundance of bacteria, and the CFU was one order of magnitude higher than that of the SPMC in this study (Fig. 8). The bacterial abundance, such as 2.97 × 10^5^ CFU ml^−1^ in the SPMC, is also comparable to that in other studies concerning phototrophic cultivation of Chlorophyta, such as the bacterial numbers of 5.5 × 10^5^ CFU ml^−1^ in a *Scenedesmus* outdoor culture [34] and 8.6 × 10^5^ CFU ml^−1^ in a *Nannochloropsis* culture [42]. The majority of these bacterial cells will be removed after biomass harvesting by settling overnight [24]. These results validate the feasibility of SPMC in enhancing oleaginous algal biomass production under open conditions.

The ACEs reported in literatures are much higher than the ACEs achieved in this study (Table 5). These studies were mainly conducted under nutrition-sufficient conditions rather than nutrition-deficient conditions. In the mixotrophic stage of SPMC, *Graesiella* cells grew on the intracellular N storage pools, which only generated a limited increase in biomass yield. Moreover, the amount of acetate supplemented was only 2.2 mM per day, which was exhausted within 24 h (Fig. 2). The limitation of both nitrate and acetate led to a relatively low ACE in the SPMC, but one that was still higher than that obtained in the nitrate-depleted stage of the phototrophic cultivation (Table 4). In this sense, the enhancing effects of SPMC can be amplified by optimizing the readily available concentrations of nitrate and acetate according to the intrinsic nutrition assimilation characteristics of microalgae.

**Table 5:** Microalgal acetate conversion efficiencies reported in literature

Cultures	Microalgae	Operation mode	ACE (g g^−1)^	Refs.
200 m^2^ SPMC (2014)	*Graesiella*	Fed batch	0.15 ± 0.08^a^	This study
200 m^2^ SPMC (2015)	*Graesiella*	Fed batch	0.21 ± 0.04^a^	This study
1000 m^2^ SPMC (2016)	*Graesiella*	Fed batch	0.21 ± 0.05^a^	This study
Heterotrophic	*Chlorella*	Chemostat	0.48	[45]
Heterotrophic	*Chlamydomonas*	Chemostat	0.48	[46]
Mixotrophic	*Chlamydomonas*	Batch	0.33–0.56	[47]

^a^Data are presented as means of three cultivations ± SD. For details of the calculation see Additional file 6

The SPMC method can be applied to mass cultivation of microalgae in a single fed-batch mode (as demonstrated in this study) to produce certain metabolites, especially those involved in nitrogen stress, such as TAG, astaxanthin, and β-carotene. Theoretically, this method could also be operated in a periodic manner by harvesting part of the lipid-rich cell mass at the end of the previous fed-batch cultivation and replenishing nitrate and phosphate to start a new fed-batch cultivation. In this case, recycling of culture medium and control of predators are the key issues to establish such a long-term cultivation system. However, achievement of the periodic fed-batch cultivation needs further study.

## Conclusions

In this study, a window period for mixotrophic cultivation of the microalga *Graesiella* sp. WBG-1 was identified during its phototrophic growth by comparing the growth rates and acetate conversion efficiencies at different growth stages. Using this period, a new sequential phototrophic–mixotrophic cultivation (SPMC) method that enhances algal biomass productivity while limiting bacteria contamination was established for the cultivation of oleaginous microalgae in open raceway ponds. The successful scale-up of SPMC in a commercial raceway pond (1000 m^2^ culture area) was demonstrated for the first time. A 56.6% increase in biomass productivity and comparable lipid content were achieved in comparison to previously reported phototrophic cultivations. The SPMC method is cost-effective and very efficient, and is able to mass produce lipid-rich Chlorophyta mixotrophically in open raceway ponds. This method is quite an attractive proposal for global producers of not only lipid-rich microalgae biomass, but also high value chemicals, such as astaxanthin and β-carotene from microalgae.

## Additional files

**Additional file 1.** Fatty acid composition of *Graesiella* sp. WBG-1 under different acetate feeding strategies and photo of the two types of column reactor.

**Additional file 2.** Cultivation of *Graesiella* sp. WBG-1 under SPMC regime at Jul 4–18/2014 in a 200 m^2^ raceway pond.

**Additional file 3.** Changes of residual nitrate and phosphate during the *Graesiella* cultivation in open raceway pond.

**Additional file 4.  ** Thin-layer chromatography of the lipids extracted from *Graesiella* sp. WBG-1.

**Additional file 5.** Correlation of light intensity and biomass productivity of the microalgae *Graesiella* sp. WBG-1 phototrophically cultured in raceway pond.

**Additional file 6.** Calculation of acetate conversion efficiency.

## Abbreviations

OD_540_: optical density at 540 nm
E-FB: fed-batch supplement of acetate during the whole culture period
A-FB: fed-batch supplement of acetate before nitrate depletion
P-FB: fed-batch supplement of acetate after nitrate depletion
A-B: batch supplement of acetate before nitrate depletion
P-B: batch supplement of acetate after nitrate depletion
DW: dry weight
*C*_biomass_: biomass concentration
*C*_lipid_: lipid content
*P*_biomass_: biomass productivity
*P*_lipid_: lipid productivity
TC: biomass carbon content
CFU: bacterial colony-forming unit
*I*_av_: average daily light intensity
*µ*: growth rate
ACE: acetate conversion efficiency
SPMC: sequential phototrophic–mixotrophic cultivation
