# Supplementary material for: Sequential phototrophic–mixotrophic cultivation of oleaginous microalga Graesiella sp. WBG-1 in a 1000 m2 open raceway pond
Source: Biotechnol Biofuels. 2019 Feb 11;12:27. doi: 10.1186/s13068-019-1367-1 (PMC6371596; doi:10.1186/s13068-019-1367-1)
Supplement: Supplementary file 2 — Additional file 2. Cultivation of Graesiella sp. WBG-1 under SPMC regime at Jul 4–18/2014 in a 200 m2 raceway pond. [file 13068_2019_1367_MOESM2_ESM.pptx]

## Slide 1
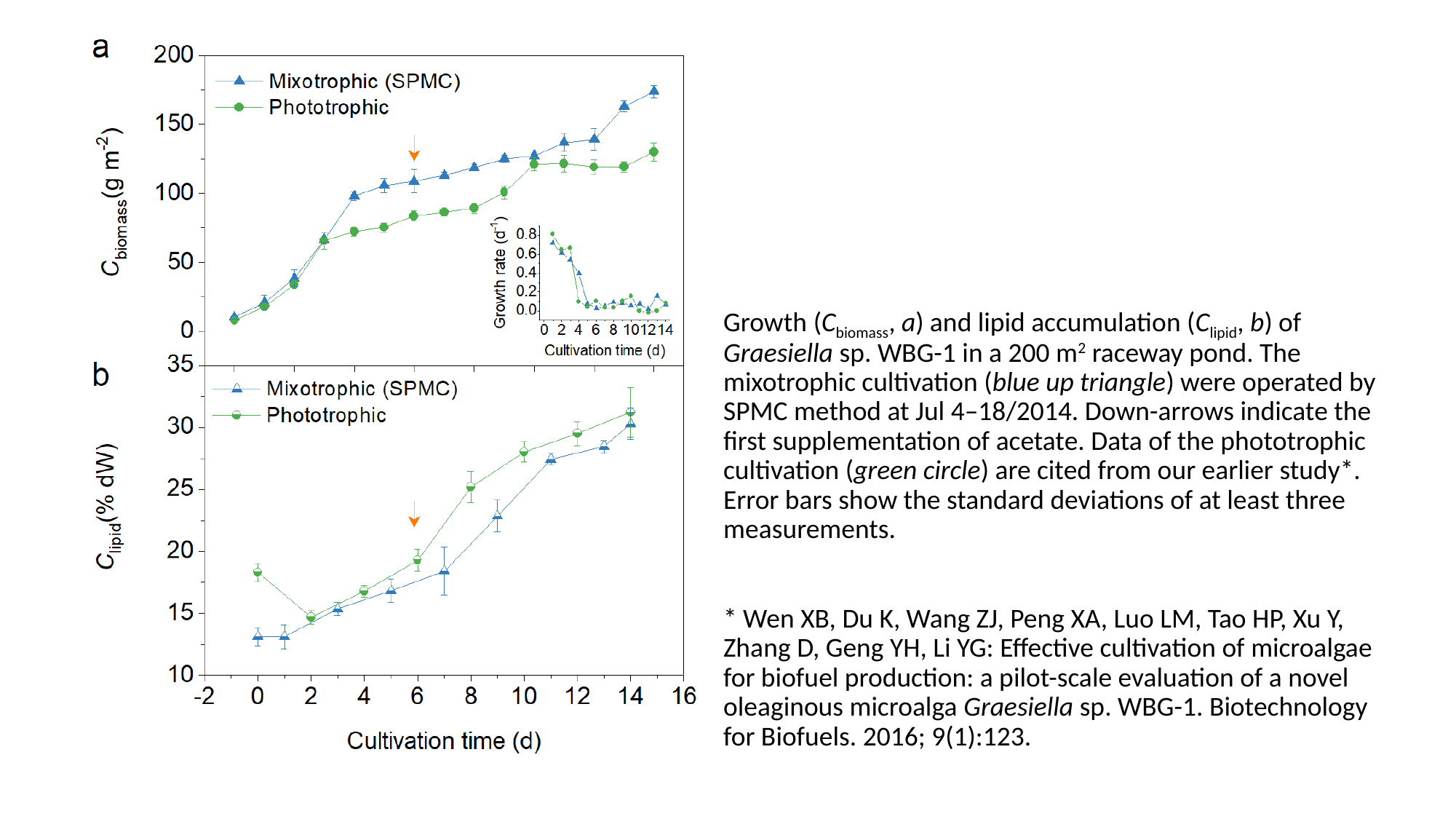

Growth (Cbiomass, a) and lipid accumulation (Clipid, b) of Graesiella sp. WBG-1 in a 200 m2 raceway pond. The mixotrophic cultivation (blue up triangle) were operated by SPMC method at Jul 4–18/2014. Down-arrows indicate the first supplementation of acetate. Data of the phototrophic cultivation (green circle) are cited from our earlier study*. Error bars show the standard deviations of at least three measurements.
* Wen XB, Du K, Wang ZJ, Peng XA, Luo LM, Tao HP, Xu Y, Zhang D, Geng YH, Li YG: Effective cultivation of microalgae for biofuel production: a pilot-scale evaluation of a novel oleaginous microalga Graesiella sp. WBG-1. Biotechnology for Biofuels. 2016; 9(1):123.
