# Supplementary material for: Sequential phototrophic–mixotrophic cultivation of oleaginous microalga Graesiella sp. WBG-1 in a 1000 m2 open raceway pond
Source: Biotechnol Biofuels. 2019 Feb 11;12:27. doi: 10.1186/s13068-019-1367-1 (PMC6371596; doi:10.1186/s13068-019-1367-1)
Supplement: Supplementary file 4 — Additional file 4. Thin-layer chromatography of the lipids extracted from Graesiella sp. WBG-1. [file 13068_2019_1367_MOESM4_ESM.pptx]

## Slide 1
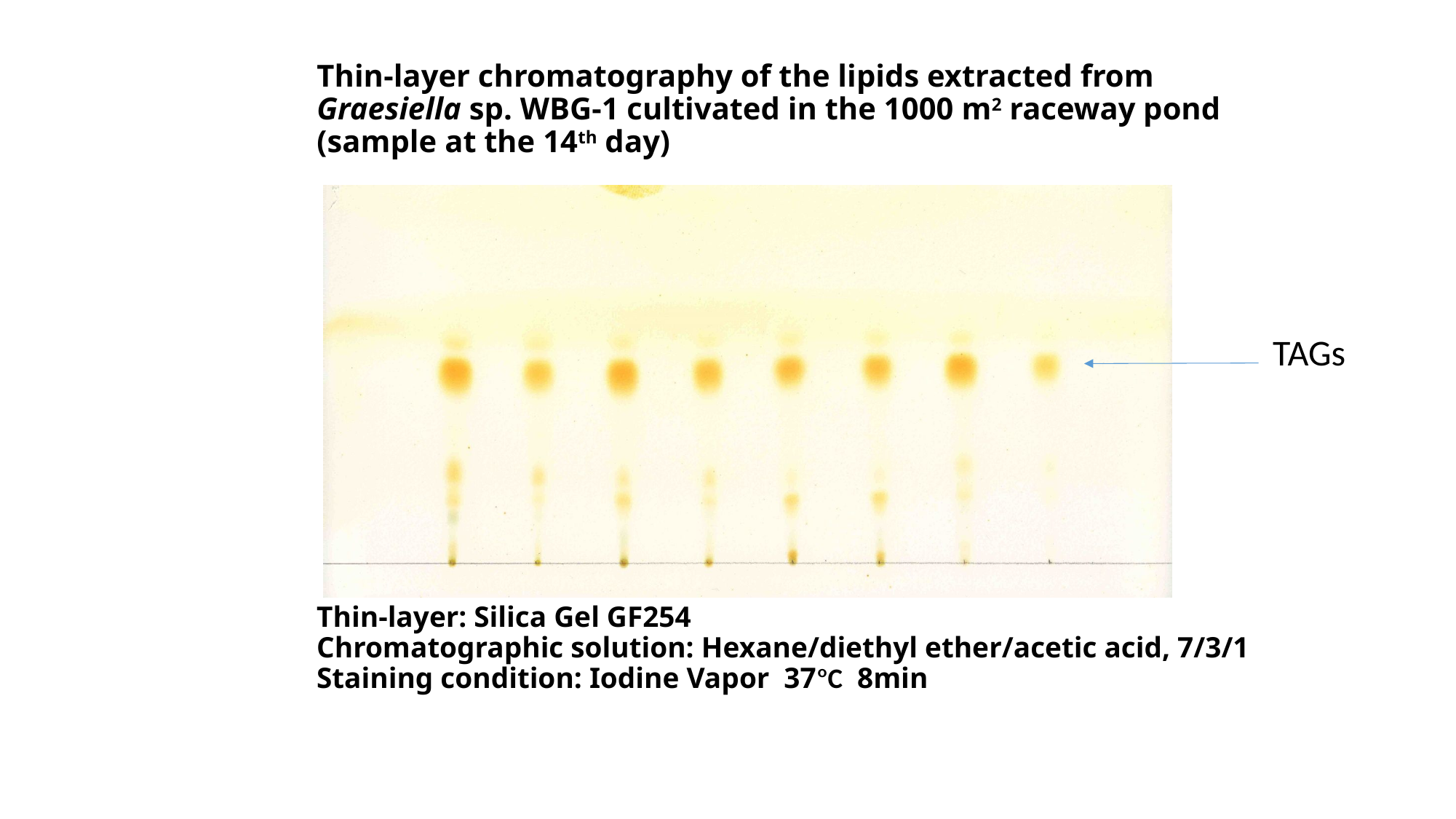

# Thin-layer chromatography of the lipids extracted from Graesiella sp. WBG-1 cultivated in the 1000 m2 raceway pond (sample at the 14th day)
TAGs
Thin-layer: Silica Gel GF254
Chromatographic solution: Hexane/diethyl ether/acetic acid, 7/3/1
Staining condition: Iodine Vapor 37°C 8min
